# Impact of foot progression angle modification on plantar loading in individuals with diabetes mellitus and peripheral neuropathy

**Published:** 2016-03-26

**Authors:** Ericka N. Merriwether, Mary K. Hastings, Kathryn L. Bohnert, John H. Hollman, Michael J. Strube, David R. Sinacore

**Affiliations:** 1 Neurobiology of Pain Laboratory, Department of Physical Therapy and Rehabilitation Science, University of Iowa, 3148 Medical Laboratories, Iowa City, IA USA 52242; 2 Applied Kinesiology Laboratory, Program in Physical Therapy, Washington University School of Medicine St. Louis, 4444 Forest Park Blvd., Campus Box 8502, St. Louis, MO USA 63108-2212; 3 Applied Kinesiology Laboratory, Program in Physical Therapy, Washington University School of Medicine St. Louis, 4444 Forest Park Blvd., Campus Box 8502, St. Louis, MO USA 63108-2212; 4 Physical Therapy Doctoral Program, Mayo School of Health Sciences, Siebens Building, Fifth Floor, 200 First St. SW, Rochester, MN USA 55905; 5 Department of Psychology, Washington University in St. Louis, Psychology Building, Room 221, Campus Box 1125, St. Louis, MO, USA 63130-4899; 6 Applied Kinesiology Laboratory, Program in Physical Therapy, Washington University School of Medicine St. Louis, 4444 Forest Park Blvd., Campus Box 8502, St. Louis, MO USA 63108-2212

**Keywords:** Diabetic foot, Gait, Peripheral neuropathy, Plantar pressure

## Abstract

**Aims:**

To determine if participants can reduce foot progression angle (FPA), and if FPA reduction decreases regional plantar stresses and forces in individuals with diabetes.

**Methods:**

**Design**

Three-group cross-sectional design with repeated measures.

**subjects**

twenty-eight participants either with diabetes mellitus (DM), diabetes and peripheral neuropathy with (DMPN+NPU) or without a prior history of ulceration (DMPN−NPU) were studied.

**Intervention**

Participants were first instructed to walk over a 3.6 m walkway at their preferred FPA, and then to walk with their foot aligned parallel with the line of gait progression at their self-selected speed. Dynamic plantar kinetics in six masked regions were collected using an EMED-st-P-2 pedobarograph.

**Main measures**

Primary outcome measures were FPA, peak plantar pressure (PPP), and force-time integral (FTI). A repeated measures ANOVA was conducted to determine group differences in FPA for both walking conditions. Regional differences in PPPs and FTIs between preferred and corrected walking conditions were analyzed using repeated measures ANCOVA.

**Results:**

Participants showed a reduction in FPA magnitude on the ‘Involved’ foot between the preferred and corrected walking conditions (*p*<0.01). There were no differences in PPPs or FTIs in any mask between walking conditions (*p*>0.05).

**Conclusion:**

Results from this investigation offer important evidence that people with diabetes can modify their FPA with a simple intervention of visual and verbal cueing. Future research should examine if gait retraining strategies in regular footwear more effectively offload areas of elevated regional plantar stresses and forces in adults with diabetes mellitus and peripheral neuropathy.

## Introduction

Elevated regional plantar stress is an index of dermal injury risk in adults with diabetes mellitus and peripheral neuropathy (DMPN), and is thought to initiate an impairment cascade of neuropathic plantar ulcer (NPU) development and subsequent non-traumatic lower extremity amputation [1, 2]. Foot progression angle (FPA), or “toe-out angle”, is an established predictor of elevated regional plantar stresses and loads in individuals with DMPN [1, 3–5]. The FPA is greater in individuals with DMPN with and without a history of NPU compared to individuals without diabetes or foot pathology [1, 2, 4]. An estimated 12–25% of individuals with DMPN have a lifetime risk of developing NPUs in the United States [6, 7]. Further, more than 65,000 non-traumatic lower extremity amputations in adults with DMPN occur annually in the United States, with 84% preceded by the development of a NPU [8, 9]. Therefore, the development and recurrence of NPUs represent a significant national economic healthcare burden.

Mueller et al. reported that FPA predicts up to 15% of the variance in medial and lateral forefoot peak plantar pressure (PPP) on the involved foot of individuals with DMPN having a prior history of NPUs [1]. Hastings and colleagues also observed FPA accounted for 35–45% of the variance in medial plantar loading in adults with DMPN with a prior history of NPUs [3]. These findings suggest FPA contributes to elevated regional plantar stresses and forces in regions of the foot susceptible to NPU development. Orthotic treatment strategies effectively offload areas of plantar ulceration in the forefoot and midfoot regions in individuals with DMPN [10–12]. However, there are often barriers related to cost, patient compliance, and reimbursement [11, 13]. Therefore, other rehabilitative strategies to offload areas of the plantar surface vulnerable to ulceration in individuals with DMPN are needed.

Gait modification strategies for older adults with DMPN such as walking slower, reducing push off in late stance phase of walking by exaggerating hip flexion, or walking with a “step-to” gait pattern using a cane have been shown to reduce PPP in the forefoot. However, regional changes in plantar stresses and forces in the forefoot and midfoot as a result of these gait modifications are variable or have not been reported [14, 15]. Additionally, it is unknown whether FPA is modifiable in individuals with diabetes mellitus with or without peripheral neuropathy, or the effect of this modification on regional plantar stresses and forces. Therefore, the purposes of this study were to: 1) determine if participants with diabetes can reduce their FPA with a simple intervention of verbal and visual cueing, and 2) determine the impact of FPA reduction on regional plantar stresses and forces. We hypothesized that participants with diabetes could reduce their FPA, which would result in concomitant decreases in regional plantar stresses in the medial forefoot and midfoot.

## MATERIALS AND METHODS

### Participants

Twenty-eight individuals with diabetes with or without accompanying peripheral neuropathy and a history of NPU participated in the study, and provided written informed consent as approved by the university’s Institutional Review Board. The purpose for selecting these participants was to determine the effect of reducing FPA in a population of adults with diabetes with and without loss of protective cutaneous sensation. Peripheral neuropathy was classified based on the presence or absence of protective cutaneous sensation and vibration perception threshold (VPT). Ulcer classification was based on any prior history of NPU. Cutaneous sensation was assessed using a 5.07 (10-gram) Semmes-Weinstein monofilament at seven sites on the plantar surface of the foot. Vibration perception threshold (VPT) was measured using a 120V biothesiometer (Bio-medical Instrument Co., Newbury, OH, 44065, USA) to assess large fiber peripheral nerve function [16]. Those who were either unable to feel the 10-gram monofilament on at least one of the seven sites on the foot or were unable to perceive vibration of the biothesiometer at threshold of less than 25 V were classified as having peripheral neuropathy. A VPT >25 V is associated with incidence of foot ulceration in individuals with type 2 diabetes mellitus [17]. The combination of these tests for evidence of peripheral neuropathy has been shown to increase specificity of risk identification and disease severity without diminution of sensitivity [17]. Based on these criteria, eleven participants were classified as having diabetes only (DM), seven as having diabetes and peripheral neuropathy without a prior history of NPU (DMPN−NPU), and ten as having diabetes, peripheral neuropathy, and a prior history of NPU (DMPN+NPU). Of the DMPN+NPU participants, eight reported a history of unilateral ulceration and two reported a history of bilateral ulceration. Participants in the DMPN+NPU group reported having NPUs in the following areas: five in the forefoot, two in the midfoot, and three in the hindfoot. Participants classified as DMPN+NPU were not ulcerated at the time of testing. Those identified as non-ambulatory or with lower extremity amputations proximal to the digits were excluded from the study.

### Plantar pressure measurement

Dynamic plantar pressures were collected using an EMED-ST-P-2 pedobarograph (Novel Inc., St. Paul, MN, USA). System specifications include a sampling frequency of 50 Hz and resolution of 2 sensors/cm^2^ for a network of 2736 sensors. Participants were selected to walk under two conditions using the two-step method, which yields similar measurements of peak plantar pressure (PPP) with the mid-gait and multiple step method [18]. Participants were first asked to walk over a 3.6 m walkway at their self-selected speed and preferred FPA. Participants were then verbally directed to align their foot along the 2nd ray (representing the longitudinal axis of the foot) on a thickened black line in the floor parallel with the line of gait progression, and walk with their foot in this corrected position over the walkway at their self-selected speed. Participants were also given verbal instructions to “keep their feet turned straight” prior to practice trials. Walking speed was measured using a stopwatch over a predetermined distance, and was expressed in m/min. Participants performed three walking trials with each foot contacting the EMED platform during each condition. All participants were allowed 1–2 practice trials prior to recording.

### Data processing

#### Masking protocol

A plantar pressure map of each footstep was generated for each participant. The plantar pressure map was divided into medial and lateral vertical masks using a 50% vertical bisector approximately between the 2nd and 3rd rays and the midpoint of the heel using Percent Mask software (Novel Inc., St. Paul, MN, USA). The plantar pressure map was further divided into three horizontal regions at 33% and 63% of foot length creating masks at the hindfoot, midfoot, and forefoot. Together, the vertical and horizontal bisections of the foot created six distinct masks: the medial and lateral forefoot, the medial and lateral midfoot, and the medial and lateral hindfoot. This masking scheme was used in previous studies of the effect of FPA on timing variables in individuals with DMPN [3].

#### Foot progression angle (FPA) measurement

The FPA was calculated as the measured angle between the line of progression (a line drawn parallel to the vertical bisector of the plantar pressure map) and the line representing the anterior-posterior bisection of the foot extending from the center of the hind foot through the 2nd and 3rd rays obtained from the plantar pressure map using a 2° increment goniometer [3]. A change of ≥4° was considered a meaningful corrected change in FPA based on reported ranges of 5–9° for FPA magnitude in young and older adults [19]. The threshold of ≥4° was, therefore, the desired response to visual and verbal cues with several practice trials. The FPA magnitude was the primary variable of interest.

#### Plantar pressure and force variables

Variables of interest were peak plantar pressure (PPP) and force-time integral (FTI), which have been used in previous work to operationally define plantar stress and force, respectively, in individuals with DMPN [10]. The PPP is the peak pressure recorded within a mask region during stance phase of the gait cycle [10]. It has been accepted as an index of risk for dermal injury on the foot plantar surface because elevated regional PPP values occur at areas of skin breakdown in individuals with diabetes that have a lack of protective sensation and a history of NPU [1]. The FTI is a description of force expressed as a calculated sum of the product of vertical force recorded from each sensor multiplied by the area and contact time of each sensor (∑(force × area × time)) for each region of the plantar surface of the foot [10]. The FTI is an accepted measure of plantar loading in individuals with DMPN representing the combined magnitude of load over the time the load is applied in each mask.

## STATISTICAL ANALYSES

Prior to all analyses, a Shapiro-Wilk test of normality was conducted to verify continuous data were normally distributed. Participants’ feet were defined as ‘Involved’ based on the foot of the DMPN+NPU group with an ulcer history. If DMPN+NPU participants had a history of bilateral involvement, the foot with the most recent ulceration was classified as the ‘Involved’ foot. The ‘Involved’ foot was randomly assigned for participants in the DM and DMPN−NPU groups. Ratios for right versus left feet classified as ‘Involved’ were analyzed using chi-square analysis. An a priori power analysis was conducted to determine the number of participants required to detect at least a 4° change in FPA between walking conditions.

### Foot progression angle (FPA)

A repeated measures analysis of variance (ANOVA) was conducted to determine group differences in FPA for both walking conditions. The between-groups factor was group assignment (DM, DMPN−NPU, DMPN+NPU), and walking condition the repeated measures factor (preferred versus corrected FPA).

### Plantar pressure and force variables

PPP and FTI for the ‘Involved’ foot for all participants were averaged over three trials, and analyzed using a repeated measures analysis of covariance (ANCOVA) with average walking speed (mean walking speed = 49 m/min) used as a covariate to account for the established influence of walking speed on PPP, as well as for the between-group differences in walking speed [4]. The between-groups factor was group assignment. Repeated measures factors were walking condition (preferred FPA versus corrected FPA), mediolateral mask location (medial versus lateral), and anteroposterior mask location (hindfoot versus midfoot versus forefoot). Post-hoc analyses for main and interaction effects were conducted using the Bonferroni correction for multiple comparisons, with statistical significance for all analyses set at *p*<0.05. Statistical analyses were performed using IBM SPSS Statistics software, version 21.0 (SPSS Inc, Chicago, IL, USA).

## RESULTS

### Participant characteristics

The mean ± SD age for all participants (N = 28) was 58±2 years. There were no group differences in age, height, or body mass index (BMI) (Table 1). The peripheral neuropathy groups (DMPN−NPU, DMPN+NPU) had greater vibration perception thresholds on the ‘Involved’ foot than the DM group, confirming the presence of peripheral neuropathy. The DMPN+NPU group had longer disease duration than the DM and DMPN−NPU groups. There were group differences in walking speed, with the DMPN+NPU group walking slower than the other diabetes groups under both conditions (Table 1). There was no difference in walking speed between conditions for either group (data not shown). Additionally, there was no group difference in the proportion of right versus left feet that were classified as ‘Involved’ (χ^2^ = 0.25, df = 2, *p* = 0.88).

### Foot Progression Angle (FPA)

There was a significant reduction in FPA magnitude on the ‘involved’ foot between the preferred and the corrected walking conditions for the combined group (*p*<.01). When assessing for group differences, the DM group showed a significant reduction in FPA between conditions compared to the DMPN+NPU group. There were no differences in FPA in either walking condition between the DMPN−NPU and the other groups. Values are given in Table 1.

### Peak Plantar Pressure (PPP)

There were no group differences in PPP in either mask (p = 0.22), nor was there a statistically significant interaction effect of walking speed, condition, and mask location (p = 0.20). Therefore, groups were combined and analyzed as a single group to determine interaction effects of condition (preferred versus corrected FPA), mediolateral mask location (medial versus lateral), and anteroposterior mask location (hindfoot versus midfoot versus forefoot). Values for PPP in each masked region for the combined group are given in Table 2. There was no difference in PPP between the preferred and corrected FPA walking conditions in either mask (p = 0.41). There was a statistically significant interaction effect of anteroposterior mask location for the combined group, with PPP of the forefoot exceeding both PPP in the midfoot (−32 N/cm^2^, *p*<0.01) and hindfoot (−18 N/cm^2^, p<.01) irrespective of walking condition. There was an increase in PPP in the medial and lateral midfoot masks (3 N/cm^2^, 5 N/cm^2^). In the forefoot masks, there was a reduction in PPP in medial forefoot (5 N/cm^2^) with a concomitant increase in the lateral forefoot (3 N/cm^2^). None of the changes in PPP in the mediolateral mask locations were statistically significant.

### Force-time Integral (FTI)

There were no group differences in FTI in either mask (p = 0.86), nor was there a statistically significant interaction effect of walking speed, condition, and mask location (p = 0.26). Therefore, groups were combined and analyzed as a single group to determine interaction effects of condition (preferred versus corrected FPA), mediolateral mask location (medial versus lateral), and anteroposterior mask location (hindfoot versus midfoot versus forefoot). Values for FTI in each mask region for the combined group are given in Table 3. There was no difference in regional FTI between the preferred and corrected FPA walking conditions (*p* = 0.21). In the hindfoot masks, there were decreases in FTI in the medial and lateral hindfoot (3 N/s, 7 N/s). There were increases in FTI in the medial and lateral midfoot masks (10 N/s, 14 N/s). In the forefoot masks, there was a slight reduction in FTI in medial forefoot (5 N/s) with a concomitant increase in the lateral forefoot (2 N/s). None of these changes was statistically significant.

## DISCUSSION

A key finding from this investigation is all participants with diabetes achieved a significant reduction in FPA magnitude in the corrected walking condition. This observation offers evidence that individuals with and without DMPN can modify FPA with a simple intervention of visual and verbal cueing, irrespective of lower extremity sensory input. However, despite significant reductions in FPA in the corrected position, there were no significant concomitant changes in regional plantar stresses or forces in the forefoot, midfoot, or hindfoot. These findings suggest modification of FPA alone may not be an effective rehabilitative strategy for reducing plantar stresses and forces in adults with diabetes. To our knowledge, this is the first study to assess the impact of FPA modification on regional plantar loading in participants with diabetes with and without peripheral neuropathy and history of NPU.

The primary purpose of the current study was to determine if participants with diabetes were able to reduce the magnitude of their FPA (“in-toeing”) with visual and verbal cueing. Participants were able to reduce their FPA by an average of 12°, greater than the clinically meaningful change of 4°. Previous studies have also sought to determine the feasibility of modifying FPA in other adult populations. Rosenbaum showed that young, healthy adults were able to modify their FPA with an induced 25° decrease (“in-toeing”) and 27° increase (“out-toeing”) in FPA in a pilot study [20]. By contrast, the magnitude of FPA reduction for participants with diabetes in the current study was well below the reported values for young, healthy participants. Individuals with DMPN have decreased hip range of motion compared with healthy adults without diabetes, which may influence the range of motion necessary to achieve a similar reduction in FPA [21]. Results from these investigations indicate that FPA reduction is achievable in adults with and without diabetes though the magnitude of the change may be population specific.

The secondary purpose of this study was to assess changes in plantar stresses and forces after FPA reduction in participants with diabetes and with or without accompanying peripheral neuropathy and a history of NPU. FPA reduction yielded a modest 3–8% decrease in medial forefoot PPPs and FTIs, with accompanying 6–17% increases in lateral forefoot and midfoot PPPs and FTIs. In a similar study conducted in young, healthy adults, Rosenbaum reported 42–46% decreases in medial forefoot and 9–22% in medial midfoot PPP and FTI as a result of an FPA reduction of at least 25° [20]. Additionally, there were concomitant 33–61% increases in lateral forefoot and midfoot PPPs and FTIs [20]. These discrepant findings may be explained in part by differences in the magnitude of the induced reductions in FPA. Furthermore, there may have been undetected fixed structural foot deformities and plantar soft tissue changes that could have precluded achieving significant reduction of regional plantar stresses and forces observed in previous studies [1, 22, 23].

Results from prior studies suggest that structural deformities, skin material properties, and shear stresses may significantly modulate the impact of FPA modification on reduction of regional plantar stresses in individuals with DMPN. Limited range of motion of the ankle, hallux valgus of the 1st metatarsophalangeal joint, and hyperextension of the metatarsophalangeal joints of the lesser toes are correlated with elevated forefoot plantar stresses, and account for up to 45% of variance in forefoot PPP in adults with DMPN [1, 24]. Also, limited metatarsophalangeal joint extension and malleolar valgus index, a measure of foot structure, account for up to 20% of plantar stresses under the forefoot in adults with DMPN [23]. In the current study, there is heterogeneity of reported NPU location in the DMPN+NPU group which may be indicative of the onset of rigid structural foot deformities. Therefore, the magnitude of FPA reduction for participants with DMPN and a history of NPU may not have been sufficient to overcome the influence of limited range of motion and structural deformities not measured in this study. Investigators have also noted increased soft tissue stiffness under metatarsal heads and higher magnitudes of subdermal shear stress during walking in adults with DMPN with a history of neuropathic plantar ulceration [22, 25, 26]. The magnitude and location of shear stresses were also not measured in this study. Thus, we cannot determine the effect of FPA reduction on other measures of barefoot plantar stress known to be elevated in individuals with DMPN with a history of NPU [26].

Other groups have studied the effect of other gait modifications on the distribution of regional plantar stresses and forces in adults with DMPN and a prior history of NPUs. Mueller et al. observed that implementing “hip flexion” and “step-to” gait modification strategies yielded 27–53% reductions in in-shoe forefoot PPP with an accompanying 24% increase in heel PPP [14, 15]. The authors, however, acknowledge these gait modifications affected movement symmetry and gait speed. Participants in this study were able to reduce their FPA without marked changes in gait speed between walking conditions. However, the changes in forefoot and hindfoot PPP as a result of modifying FPA were not as substantial as those reported by Mueller et al. One possible explanation for the difference may be the cumulative effect of the “hip flexion” and “step-to” gait modification strategies plus the offloading properties of footwear. One of the objectives of the current study was to examine the effect of modifying FPA on the distribution of regional plantar stresses and forces under barefoot walking conditions. Therefore, we cannot generalize these findings to the combined effect of FPA modification to the addition of therapeutic foot wear with in-shoe pressure measurements. Future studies should examine the impact of FPA modification on in-shoe measurements of regional plantar stresses and forces in individuals with DMPN.

Limitations associated with this study are noted to improve understanding of the clinical utility and generalizability of the findings. One of the primary limitations of this study is a small sample size. A sample size of 17 to 51 participants was needed to detect changes in PPP and in FTI in medial and lateral forefoot, midfoot, and hindfoot regions based on a post-hoc power analysis (1-β = 0.80). Though an a priori analysis was performed to detect change in FPA between conditions, the current investigation was not adequately powered to detect significant interaction effects of group, condition, and mask as indicators of shifts in PPP and FTI. In addition, FPA modification consisted of single session instruction, with measurements taken over several single steps. Future studies could expand these findings by assessing the effects of changing FPA over multiple steps, and determining the effects of modifying FPA on other parts of the lower extremity kinetic chain.

## CONCLUSION

In summary, results from this investigation offer important evidence that people with diabetes and peripheral neuropathy (DMPN) can modify their foot progression angle (FPA) with a simple intervention of visual and verbal cueing irrespective of lower extremity sensory input. However, successful reduction of FPA, a predictor of elevated plantar stress, did not yield concomitant reductions in regional plantar stresses and forces in individuals with DMPN under barefoot walking conditions. Therefore, examining the effect of FPA modification on in-shoe regional plantar stresses and forces in a larger sample of individuals with DMPN may be warranted. The FPA modification or alternative gait retraining strategies while donning regular footwear may more effectively offload areas of the foot at risk for NPU development. Furthermore, gait retraining is a simple, cost-effective therapeutic intervention that could be safely and quickly implemented in a physical therapist practice.

## Figures and Tables

**Table 1:** Participant characteristics, mean (SD)

	total (N = 28) Mean (SD)	DM (N = 11) Mean (SD)	DMPN-NPU (N = 7) Mean (SD)	DMPN+NPU (N = 10) Mean (SD)	P
Age [years]	58 (2)	58 (3)	61 (4)	57 (4)	0.80
Height [m]	2 (0.2)	2 (0)	2 (0.3)	2 (0.3)	0.70
BMI [kg/m2]	38 (2)	33 (2)	43 (2)	40 (3)	0.09
Disease duration [years]	13.8 (2)	7.4 (2)	11.3 (3)	23.5 (2)*	<0.01
Great Toe VPT [volts]	28 (3)	15 (2)	35 (5)#	36 (5)*	<0.01
Walking speed [meters/ min]	50 (13)	58 (7)	54 (6)	39 (14)*	<0.01
‘Involved’ foot (Right/ Left)	20R/8L	8R/3L	5R/2L	7R/3L	0.88
Preferred FPA [deg]	−14 (5)	−13 (4)	−13 (4)	−16 (6)	0.22
Corrected FPA [deg]	−2 (13)	4 (14)	2 (14)	−11 (8)*	0.02

**Abbreviations**: BMI: Body mass index. Great Toe VPT: vibration perception threshold at the great toe of the ‘Involved’ foot (Volts).

* : group differences in disease duration, great toe VPT, and walking speed between DM and DMPN-NPU groups versus DMPN+NPU group; difference in corrected FPA between DM and DMPN+NPU groups.

# : group differences in great toe VPT between DM versus DMPN-NPU groups, P<0.01

**Table 2:** Peak plantar pressure (PPP) on ‘Involved’ foot of participants with diabetes for both FPA walking conditions.

PPP Mask	pFPA Mean (SD)	cFPA Mean (SD)	% difference	P
Medial Forefoot (N/cm^2^)	59 (24)	54 (30)	−8	.41
Lateral Forefoot (N/cm^2^)	45 (26)	48 (27)	6	
Medial Midfoot (N/cm^2^)	9 (5)	12 (11)	18	
Lateral Midfoot (N/cm^2^)	27 (20)	32 (22)	17	
Medial Hindfoot (N/cm^2^)	34 (18)	33 (15)	−2	
Lateral Hindfoot (N/cm^2^)	33 (14)	34 (22)	4	

**Abbreviations**: Mean (SD) for peak plantar pressure (PPP) for all plantar masks. Values represent interaction effects of Condition [preferred FPA (pFPA) versus corrected FPA (cFPA)] × Mediolateral Mask (Lateral versus Medial) × Anteroposterior Mask (Hindfoot versus Midfoot versus Forefoot).

**Variable descriptions:** pFPA: PPP values for the preferred FPA walking condition; cFPA: PPP values for the corrected FPA walking condition. P: significance values for the interaction effect of condition × mediolateral mask × anteroposterior mask locations.

**Table 3:** Force-time integral (FTI) on ‘Involved’ foot of participants with diabetes for both FPA walking conditions.

FTI Mask	pFPA Mean (SD)	cFPA Mean (SD)	% difference	P
Medial Forefoot (N/s)	171 (74)	166 (81)	−3	.21
Lateral Forefoot (N/s)	119 (39)	121 (37)	2	
Medial Midfoot (N/s)	14 (17)	24 (37)	39	
Lateral Midfoot (N/s)	124 (74)	138 (76)	11	
Medial Hindfoot (N/s)	91 (47)	88 (43)	−3	
Lateral Hindfoot (N/s)	123 (61)	116 (58)	−6	

**Abbreviations:** : Mean (SD) for force-time integral (FTI) for all plantar masks. Values represent interaction effects of Condition [preferred FPA (pFPA) versus corrected FPA (cFPA)] × Mediolateral Mask (Lateral versus Medial) × Anteroposterior Mask (Hindfoot versus Midfoot versus Forefoot).

**Variable descriptions:** pFPA: FTI values for the preferred FPA walking condition; cFPA: FTI values for the corrected FPA walking condition. P: significance values for the interaction effect of condition × mediolateral mask × anteroposterior mask locations.
